# Apparent and standardized ileal amino acid digestibility of corn and wheat in 10- to 14-day-old Japanese quail using the nitrogen-free diet method

**DOI:** 10.1016/j.psj.2026.107658

**Published:** 2026-08-19

**Authors:** Mahmoud Ghazaghi, Mohammad Rokouei, Mehran Mehri

**Affiliations:** Department of Animal Sciences, College of Agriculture, University of Zabol, Zabol, 98661-5538, Iran

**Keywords:** Corn, Japanese quail, Nitrogen-free diet, Standardized ileal digestibility, Wheat

## Abstract

Accurate age-specific information on amino acid digestibility is needed for precision formulation of diets for young Japanese quail. Following our previous evaluation of corn and wheat in older quail, this study determined the apparent ileal digestibility (AID) and standardized ileal digestibility (SID) of amino acids in corn and wheat during the 10–14-day post-hatch period, representing an earlier starter phase than that evaluated previously. A total of 300 quail chicks were assigned to three treatments in a completely randomized design with five replicates of 20 birds each. Treatments consisted of a nitrogen-free diet (NFD) to estimate basal endogenous losses and corn- or wheat-based diets in which the test ingredient served as the sole source of protein and amino acids. Titanium dioxide (5 g/kg) was included as an indigestible marker. After a 5-d feeding period, ileal digesta were collected and amino acid concentrations in feed and digesta were determined by HPLC following acid hydrolysis. Basal endogenous amino acid losses ranged from 97.20 mg/kg dry matter intake (DMI) for aspartic acid to 802.78 mg/kg DMI for proline, with substantial variation among amino acids. In corn, AID and SID values ranged from 70.21 to 94.30% and from 83.96 to 97.53%, respectively, whereas corresponding values for wheat ranged from 77.96 to 90.81% and from 84.15 to 95.48%. The magnitude and direction of differences between ingredients varied among amino acids. Corn exhibited higher SID values for lysine, valine, threonine, and arginine, whereas wheat showed higher SID values for glutamic acid, glycine, leucine, histidine, alanine, and serine (P < 0.05). Although the magnitude of differences varied among amino acids, methionine exhibited relatively small differences between ingredients, whereas aspartic acid showed only moderate variation. These findings demonstrate substantial ingredient-specific variation in amino acid digestibility and emphasize the importance of using species-specific digestibility coefficients when formulating diets for growing Japanese quail.

## Introduction

Protein is one of the most costly components of poultry diets, and accurate formulation based on digestible amino acids (AA) rather than total amino acids is essential for optimizing performance and reducing nitrogen excretion (Lemme et al., 2004). Standardized ileal digestibility (SID) has become the preferred system for evaluating AA availability in poultry. The SID system corrects apparent ileal digestibility (AID) for basal endogenous losses (BEL) and therefore provides a more biologically meaningful estimate of nutrient utilization. In poultry nutrition, SID values are widely used in feed formulation of broiler chickens because they improve precision in meeting AA requirements and support more efficient use of feed ingredients (Adedokun et al., 2008; Adedokun et al., 2007; Garcia et al., 2007).

Corn and wheat are among the most important cereal ingredients in poultry diets, but their AA digestibility can differ because of variations in protein composition, fiber content, and other chemical characteristics. Previous studies in broiler chickens have shown that SID of AA can vary substantially among ingredients and that nutrient composition may be useful in predicting digestibility coefficients (Szczurek et al., 2020; Yun et al., 2023). These findings highlight the importance of generating ingredient‑specific digestibility values rather than relying on generalized feed composition tables (Vasan et al., 2008).

Although most digestibility research has been conducted in broilers, there is increasing interest in species‑specific nutrient evaluation for Japanese quail (Ahmadi et al., 2025; Heydari et al., 2025). Japanese quail may differ from broiler chickens in growth pattern, digestive physiology, and amino acid utilization, so amino acid digestibility values derived from broilers may not be directly applicable to quail diets. Therefore, digestibility coefficients generated in broilers or other poultry species cannot be assumed to apply directly to quail (Vasan et al., 2008).

The nitrogen‑free diet (NFD) method is commonly used to estimate basal endogenous amino acid losses in poultry, and it has been applied successfully in studies of standardized ileal digestibility (Adedokun et al., 2011). This approach is particularly valuable in young birds, where endogenous secretions can contribute substantially to ileal amino acid flow and bias apparent digestibility estimates. Accurate estimation of BEL is therefore a necessary step in developing reliable SID coefficients for quail (Ghazaghi et al., 2023). Despite the growing importance of Japanese quail production, information on SID coefficients of AA in this species remains limited compared with broiler chickens. In a previous study, Ghazaghi et al. (2023) determined the apparent and standardized ileal amino acid digestibility of corn and wheat, as well as other feed ingredients, in Japanese quail at 14–18 and 28–32 days of age. That study demonstrated that amino acid digestibility varies with both ingredient type and bird age. However, amino acid digestibility during the earlier 10–14-day post-hatch period was not evaluated. Because this period represents an earlier stage of gastrointestinal development and nutrient utilization, digestibility coefficients obtained from older quail may not necessarily represent amino acid utilization during the early starter period.

Thus, the present study was designed as an age-specific extension of our previous work to determine the apparent and standardized ileal digestibility of amino acids in corn and wheat during the 10–14-day post-hatch period. The objective was not to re-evaluate the general digestibility of corn and wheat in Japanese quail, but to provide digestibility coefficients specifically for the early starter period and to compare these values with those previously reported for older quail.

## Materials and methods

### Bird management

The experimental protocol was approved by the Research Animal Ethics Committee of the University of Zabol (approval code: AECUZ-2012-BR-2025-24) and the Iranian Council of Animal Care. A total of 300 quail chicks were randomly allocated to 15 floor pens, with 20 birds per pen. The pens were then randomly assigned to three dietary treatments, resulting in five replicate pens per treatment. Thus, the pen was considered the experimental unit. Chicks received a standard grower diet based on NRC (2026) recommendations until day 9 post hatch. From day 10 to day 14 (a 5‑day experimental period), birds were fed the experimental diets: a nitrogen‑free diet (NFD), a wheat‑based diet, or a corn‑based diet. On day 14, all birds were euthanized using carbon dioxide (CO₂) inhalation in accordance with the approved animal-care protocol, and ileal contents were subsequently collected. During the experimental period (10–14 days post hatch), the housing temperature was maintained at 30°C with a relative humidity of 60%. A lighting schedule of 16 h light: 8 h dark was applied throughout the study. Birds had *ad libitum* access to feed and water.

### Experimental diets

All feed ingredients were analyzed for crude protein and amino acid profiles prior to feed formulation (Table 1). The nitrogen‑free diet (NFD) was a semi‑purified diet based on cornstarch, used to determine BEL of amino acids. The wheat‑based and corn‑based diets were formulated such that wheat or corn served as the sole source of crude protein and amino acids (Table 2) as earlier reported by Ghazaghi et al. (2023). Titanium dioxide (5 g/kg of diet) was added to all diets (mash form) as an indigestible marker.

**Table 1:** Amino acid content of ingredients.

Amino acid (%)	Corn	Wheat
Lys	0.24	0.35
Met	0.16	0.19
Cys	0.18	0.27
Thr	0.29	0.35
Trp	0.06	0.14
Arg	0.38	0.59
Ile	0.27	0.41
Leu	0.96	0.79
Val	0.37	0.52
His	0.23	0.28
Phe	0.39	0.53
Asp	0.53	0.64
Glu	1.45	3.26

Alanine (Ala), Arginine (Arg), Aspartic acid (Asp), Glutamic acid (Glu), Glycine (Gly), Histidine (His), Isoleucine (Ile), Leucine (Leu), Lysine (Lys), Methionine (Met), Proline (Pro), Serine (Ser), Threonine (Thr), and Valine (Val).

**Table 2:** Composition of the experimental diets.

Ingredient (%)	N-free diet	Corn	Wheat
Test ingredient	-	80.0	88.0
Corn starch	66.26	16.33	7.30
Sucrose	24.30	-	-
Solka-floc	5.00	-	-
Dicalcium Phosphate	1.50	-	1.39
K_2_CO_3_	1.36	0.36	-
NaHCO_3_	-	0.50	-
TiO_2_	0.50	0.50	0.50
Vitamin premix^1^	0.25	0.25	0.25
Mineral premix^2^	0.25	0.25	0.25
NaCl	0.38	0.20	0.31
MgO	0.10	0.10	1.00
Choline chloride	0.10	0.10	1.00
CaCO3	-	1.41	-
Calculated composition			
AME (kcal/kg)	3410	3400	2830
Crude protein (%)	-	6.34	12.32
Ca (%)	0.80	1.00	1.00
Na (%)	0.15	0.23	0.16
K (%)	0.60	0.40	0.40
Cl (%)	0.23	0.15	0.23
DEB^3^ (mEq/kg)	210.0	118.6	242.1
Crude fiber (%)	4.95	1.76	2.64
NSP^4^ (%)	-	7.55	10.60

1 Mineral premix provided per kilogram of diet: Mn, 100 mg; Se, 0.2 mg; I, 1.0 mg; Cu, 100 mg; Fe, 50 mg.

2 Vitamin premix provided per kilogram of diet: vitamin A, 11,000 IU; cholecalciferol, 2,300 IU; vitamin E, 121 IU; vitamin K_3_, 2.0 mg; vitamin B_12_, 0.02 mg; thiamin, 4.0 mg; riboflavin, 4.0 mg; folic acid, 1.0 mg; biotin, 0.03 mg; pyridoxine, 4.0 mg; choline chloride, 840 mg; ethoxyquin, 0.125 mg.

3 DEB: Dietary electrolyte balance

4 NSP: Non starch polysaccharides

### Digesta sampling

Ileal digesta (collected from Meckel’s diverticulum to approximately 1 cm proximal to the ileo‑cecal junction) were gently flushed with distilled water into plastic containers. Samples from each pen were pooled, frozen, and stored at −20°C until processing. Subsequently, samples were freeze‑dried, ground with a mortar and pestle, and submitted for AA and titanium analysis.

### Chemical analysis

Feed samples were analyzed for AA composition according to the procedures previously described by Ghazaghi et al. (2023). Briefly, samples were hydrolyzed for 24 h in 6 N hydrochloric acid at 110°C under a nitrogen atmosphere following AOAC (2006) procedures. Methionine and cysteine were determined after performic acid oxidation prior to acid hydrolysis, whereas tryptophan was analyzed following alkaline hydrolysis with barium hydroxide. Amino acid separation and quantification were performed using a Waters HPLC system (Waters, Milford, MA) equipped with a 1525 Binary HPLC pump, a 2487 Dual λ absorbance detector (254 nm), Breeze chromatography software, a Rheodyne 7725 injection valve (20 μL sample loop), and a PicoTag column (3.9 × 150 mm, 5 μm particle size). Titanium dioxide concentrations were determined after sulfuric acid hydrolysis using the colorimetric procedure described by Short et al. (1996).

### Calculations

The BEL, AID, and SID coefficients of AA were calculated according to Adedokun et al. (2008):

$$BEL\;\left(mg/kg\;DMI\right)\;=\;AA_{digesta}\;\times \;\frac{Ti_{diet}}{Ti_{digesta}}$$

$$AID\;\left(\%\right)=\;\left[1-\;\left(\frac{Ti_{diet}}{Ti_{digesta}}\times \frac{AA_{digesta}}{AA_{diet}}\right)\times \;100\right]$$

$$SID\;\left(\%\right)=AID+\;\left[\frac{BEL}{AA_{diet}}\times \;100\right]$$

where AA_diet_ and AA_digesta_ represent the amino acid concentrations in the diet and ileal digesta, respectively; Ti_diet_ and Ti_digesta_ represent titanium dioxide concentrations in the diet and ileal digesta, respectively; and BEL represents the basal endogenous loss of the corresponding amino acid expressed per kilogram of dry matter intake (DMI).

### Statistical analysis

Data were analyzed as a completely randomized design using feed type (corn *vs.* wheat) as the fixed effect. Each treatment consisted of five replicate pens with 20 birds per pen, and the pen served as the experimental unit. Least squares means (LSMeans) and standard errors of the mean (SEM) were calculated for each AA for both AID and SID. Differences between feed ingredients were evaluated using analysis of variance, and pairwise comparisons of LSMeans were performed using Tukey’s honestly significant difference (HSD) test to control for multiple comparisons. Significance was declared at *P* < 0.05. Results are presented as LSMeans ± SEM, together with the corresponding *P*-values. All statistical analyses were conducted using R version 4.6.1 (R Core Team, 2026). Data manipulation and graphical visualization were performed using the *dplyr, tidyr*, and *ggplot2* packages, whereas mean separation was conducted using the *emmeans* package with Tukey adjustment. Differences between ingredients (corn − wheat) and their corresponding 95% confidence intervals were displayed using a forest plot to facilitate comparison among amino acids.

## Results

### Basal endogenous amino acid losses

The BEL of AA determined using the NFD are presented in Table 3. Among the AA measured, leucine and isoleucine exhibited the highest endogenous losses, followed by valine and lysine. Intermediate losses were observed for threonine and arginine, whereas methionine, histidine, and proline showed comparatively lower losses, averaging 32.6, 80.1, and 15.7 mg/kg DMI, respectively.

**Table 3:** Endogenous amino acid losses (BEL) in Japanese quail aged 10–14 days.

Amino acid	BEL (mg/kg DMI)	SE
Lys	162	27.8
Ile	253	16.0
Met	162	11.6
Val	181	13.6
Thr	478	38.4
Arg	538	58.3
Glu	608	43.6
ASP	97	17.3
LUE	259	35.0
SER	405	29.4
GLY	216	18.0
HIS	412	28.7
ALA	199	20.8
PRO	803	59.3

Alanine (Ala), Arginine (Arg), Aspartic acid (Asp), Glutamic acid (Glu), Glycine (Gly), Histidine (His), Isoleucine (Ile), Leucine (Leu), Lysine (Lys), Methionine (Met), Proline (Pro), Serine (Ser), Threonine (Thr), and Valine (Val).

### Apparent ileal digestibility of amino acids

The AID coefficients of AA in corn and wheat are presented in Table 4. Most AA differed significantly between ingredients. Corn exhibited greater AID coefficients than wheat for arginine, aspartic acid, isoleucine, lysine, methionine, proline, threonine, and valine. In contrast, wheat showed higher AID values for alanine, glutamic acid, glycine, histidine, leucine, and serine. The greatest disparity between ingredients was observed for threonine, with corn exhibiting an AID coefficient nearly twice that of wheat. The particularly low threonine digestibility observed for wheat warrants emphasis. The AID and SID of threonine in wheat were 41.98 and 56.09%, respectively, compared with 80.45 and 97.34% in corn. The magnitude of this difference was substantially greater than that reported previously for wheat in Japanese quail.

**Table 4:** Apparent digestibility coefficients (%) of amino acids in corn and wheat (mean ± SE) for Japanese quail at 10–14 days of age.

AA	Corn	Wheat	*P*-value
ALA	60.56 ± 3.76	76.33 ± 0.56	< 0.001
Arg	80.22 ± 0.34	63.13 ± 1.79	< 0.001
ASP	95.64 ± 0.49	88.14 ± 0.67	0.0149
Glu	50.32 ± 4.44	93.35 ± 0.12	< 0.001
GLY	71.36 ± 2.88	82.42 ± 1.44	0.0004
HIS	55.96 ± 3.27	71.82 ± 1.35	< 0.001
Ile	89.86 ± 0.19	75.00 ± 2.04	< 0.001
Leu	73.29 ± 2.02	83.57 ± 1.80	0.001
Lys	88.45 ± 1.20	71.64 ± 2.82	< 0.001
Met	79.28 ± 1.17	62.11 ± 2.75	< 0.001
PRO	82.50 ± 2.18	73.04 ± 0.97	0.0023
SER	46.54 ± 2.31	55.46 ± 3.13	0.004
Thr	80.45 ± 1.33	41.98 ± 3.44	< 0.001
Val	93.72 ± 0.61	80.39 ± 1.23	< 0.001

Alanine (Ala), Arginine (Arg), Aspartic acid (Asp), Glutamic acid (Glu), Glycine (Gly), Histidine (His), Isoleucine (Ile), Leucine (Leu), Lysine (Lys), Methionine (Met), Proline (Pro), Serine (Ser), Threonine (Thr), and Valine (Val).

### Standardized ileal digestibility of amino acids

The SID coefficients of AA are shown in Table 5. Correction for endogenous amino acid losses increased digestibility values for both ingredients compared with their corresponding AID coefficients. Corn exhibited higher SID coefficients than wheat for arginine, aspartic acid, isoleucine, lysine, methionine, proline, threonine, and valine (*P* < 0.01). Conversely, wheat showed greater SID coefficients for alanine, glutamic acid, glycine, histidine, and leucine (*P* < 0.01). Serine digestibility did not differ between ingredients (*P* = 0.074).

**Table 5:** Standardized digestibility coefficients (%) of amino acids in corn and wheat (mean ± SE) for Japanese quail at 10–14 days of age.

AA	Corn	Wheat	*P*-value
ALA	64.06 ± 3.49	80.92 ± 0.97	< 0.001
Arg	94.50 ± 1.03	72.53 ± 0.44	< 0.001
ASP	97.44 ± 0.16	89.72 ± 0.53	0.0075
Glu	55.18 ± 4.09	95.08 ± 0.19	< 0.001
GLY	78.36 ± 2.29	86.77 ± 0.87	0.0037
HIS	72.47 ± 4.24	86.88 ± 0.33	< 0.001
Ile	98.33 ± 0.78	80.45 ± 1.66	< 0.001
Leu	75.82 ± 1.86	86.88 ± 1.33	0.0002
Lys	95.75 ± 0.96	76.39 ± 3.20	< 0.001
Met	80.31 ± 1.13	70.76 ± 2.28	0.0011
PRO	96.68 ± 1.69	80.43 ± 0.64	< 0.001
SER	58.18 ± 2.38	63.27 ± 3.10	0.0744
Thr	97.34 ± 0.59	56.09 ± 2.95	< 0.001
Val	98.41 ± 0.29	83.64 ± 1.28	< 0.001

Alanine (Ala), Arginine (Arg), Aspartic acid (Asp), Glutamic acid (Glu), Glycine (Gly), Histidine (His), Isoleucine (Ile), Leucine (Leu), Lysine (Lys), Methionine (Met), Proline (Pro), Serine (Ser), Threonine (Thr), and Valine (Val).

## Discussion

The present study extends our previous work on amino acid digestibility in Japanese quail by characterizing the apparent and standardized ileal digestibility of amino acids in corn and wheat during the 10–14-day post-hatch period. Whereas Ghazaghi, et al. (2023) evaluated these ingredients in quail at 14–18 and 28–32 days of age, the present experiment focused on the earlier starter period. Therefore, the principal contribution of the present study is the generation of age-specific digestibility information for 10–14-day-old quail rather than a first evaluation of corn and wheat digestibility in this species. The results also provide an opportunity to consider whether digestibility coefficients obtained from older quail are applicable to the earlier starter period.

The results confirm that ingredient identity strongly influences AA utilization in quail and that digestibility values derived from broiler chickens should not be directly extrapolated to quail (Vasan et al., 2008). Because feed formulation based on SID amino acids is more accurate than formulation based on total amino acids, these findings may provide useful information for improving the precision of amino acid formulation in quail diets; however, their effects on growth performance, feed efficiency, or nitrogen excretion were not evaluated in the present study and require confirmation in feeding trials.

Basal endogenous amino acid losses estimated with the NFD were substantial for several AA, particularly leucine, isoleucine, valine, lysine, and threonine. This pattern is consistent with the concept that endogenous secretions, sloughed intestinal cells, mucins, digestive enzymes, and microbial residues contribute meaningfully to ileal amino acid flow in young poultry (Adeola et al., 2016; Blok et al., 2017). The comparatively low losses observed for proline and methionine suggest that endogenous flow is AA specific and cannot be assumed to be uniform across AA. Such variation reinforces the importance of correcting apparent digestibility values for basal losses when comparing ingredients or estimating ingredient quality (Adeola et al., 2016; Teng et al., 2021). The high endogenous losses of branched-chain AA and lysine in the present study are broadly consistent with previous poultry work using NFD, which has shown that young birds often exhibit greater endogenous AA flow than older birds (Barua et al., 2021). In quail, this is especially relevant because the early post-hatch period is characterized by rapid gut development, changing mucosal turnover, and still-maturing digestive physiology. Therefore, the magnitude of basal losses in the 10- to 14-day age range likely reflects both the age of the birds and the physiological stress associated with rapid growth and adaptation to plant-based diets (Adeola et al., 2016; Barua et al., 2021; Ghazaghi et al., 2023).

Corn showed higher SID coefficients than wheat for lysine, threonine, arginine, valine, isoleucine, proline, methionine, and aspartic acid, whereas wheat performed better for glutamic acid, glycine, alanine, histidine, leucine, and serine. This AA-dependent response indicates that digestibility is driven not only by total crude protein content but also by the structure and localization of protein within the grain matrix. Differences in protein structure, kernel anatomy, and associations between proteins and non-starch polysaccharides may partly explain the observed variation in amino acid digestibility between corn and wheat (Widyaratne and Zijlstra, 2007).

The especially large advantage of corn over wheat for threonine and lysine is biologically meaningful because these AAs are among the most important limiting AAs in poultry diets. The markedly low threonine digestibility observed in the wheat-based diet requires cautious interpretation. In the present study, wheat had an AID and SID for threonine of 41.98 and 56.09%, respectively, whereas the corresponding values for corn were 80.45 and 97.34%. These differences are substantially larger than those reported previously in Japanese quail. Ghazaghi et al. (2023) reported wheat threonine AID values of 82.6 and 81.5% and SID values of 89.8 and 92.9% for quail at 14–18 and 28–32 days of age, respectively. Thus, the low threonine digestibility observed in the present wheat sample cannot be attributed to age alone, because the previous study did not show a similarly low value in older quail and reported relatively stable wheat threonine digestibility between the two age periods. Several factors could potentially contribute to the discrepancy, including differences in experimental age, dietary formulation, analytical measurements, endogenous loss estimation, and ingredient characteristics. Although the wheat amino acid composition reported in the present study was similar to that reported previously, digestibility coefficients may be influenced by factors beyond total amino acid concentration, including protein matrix characteristics and experimental conditions. Nevertheless, the present experiment was not designed to identify the specific cause of the unusually low threonine digestibility in wheat. Therefore, this result should be interpreted as a sample- and experiment-specific observation rather than as a general characteristic of wheat in young quail. Lysine is also highly relevant in formulation because it is commonly used as a reference AA for balancing ideal protein patterns (Emmert and Baker, 1997). Lysine is also highly relevant in poultry diet formulation because it is commonly used as a reference amino acid for establishing ideal protein patterns. In the present experiment, the tested corn sample had a higher SID of lysine than the tested wheat sample (95.75 vs. 76.39%). This difference indicates greater lysine digestibility for the corn sample under the experimental conditions, but should not be interpreted as a general characteristic of corn relative to wheat because only one sample of each ingredient was evaluated (Adeola et al., 2016). Wheat exhibited greater digestibility coefficients for several AA, particularly glutamic acid and glycine. These differences may reflect variations in protein composition and interactions between proteins and cell-wall components, emphasizing that AA digestibility depends on both ingredient type and AA characteristics (Ghazaghi et al., 2023; Widyaratne and Zijlstra, 2007).

The similarity between the ingredient ranking for AID and SID indicates that correction for BEL did not materially change the relative digestibility pattern between corn and wheat. This suggests that the main differences were driven by ingredient-intrinsic factors rather than by differences in endogenous flow alone. Such a finding is important because it implies that the observed differences appear to arise primarily from ingredient-related factors rather than from differences in BEL. In practical terms, the SID system still improves biological interpretation, but it does not eliminate the underlying compositional differences between ingredients (Adeola et al., 2016).

Compared with previous quail data, the present values appear consistent with the notion that digestibility in quail may exceed or differ from that in broilers depending on ingredient and age. Species differences in digestive physiology and age-related changes in gut development may contribute to variation in digestibility values between quail and broilers. This may partly explain the pronounced ingredient-by-amino-acid variation observed in the present experiment (Adeola et al., 2016; Ghazaghi et al., 2023). From a formulation perspective, the data suggest that corn and wheat should not be treated as nutritionally interchangeable protein carriers in young Japanese quail. For AAs such as lysine, threonine, and valine, corn appears to provide more favorable digestibility, whereas wheat may be advantageous for glutamic acid, glycine, alanine, and leucine. Because diet formulation is typically driven by the most limiting AAs, the lower digestibility of threonine and lysine in wheat is particularly relevant and may have important implications for balancing starter diets in quail. From a formulation perspective, the present data may be useful for developing diets based on digestible amino acids; however, whether the use of these ingredient-specific SID coefficients improves growth performance, feed efficiency, or reduces nitrogen excretion was not determined in this experiment and requires validation in subsequent feeding studies (Alabi and Adedokun, 2025).

A noteworthy outcome of the study is the relatively large difference between ingredients for threonine and glutamic acid. Threonine is often sensitive to intestinal health and mucin turnover, which may help explain why its digestibility differed so strongly between corn and wheat. The glutamic acid response likely reflects the predominance of wheat gluten proteins and the high abundance of glutamic acid in cereal protein fractions. Together, these findings indicate that individual AAs respond differently to ingredient matrix effects, making it inappropriate to infer the digestibility of one AA from another or to rely on crude protein digestibility as a proxy for ingredient quality (Alabi and Adedokun, 2025; Pesti and Choct, 2023).

The present study also has practical implications for quail nutrition research and feed evaluation. First, it supports the use of NFD to estimate BEL in young quail, while recognizing that the choice of method can influence the magnitude of endogenous flow. Second, it demonstrates that starter quail require ingredient-specific digestibility data rather than values borrowed from broilers or older quail. Third, it highlights the need for more comprehensive digestibility databases covering additional feed ingredients, ages, and genetic strains of quail (Adeola et al., 2016; Teng et al., 2021). There are also limitations that should be acknowledged. The study evaluated only two cereal ingredients and one early age window, so the results cannot be generalized to all feedstuffs or growth stages. In addition, the use of a nitrogen-free diet may over- or under-estimate true basal endogenous losses for certain amino acids under some conditions, and methodological differences between laboratories can affect comparability. Finally, the work did not assess performance responses, so the biological consequences of the observed digestibility differences should be confirmed in subsequent feeding trials (Adeola et al., 2016).

**Fig. 1:**
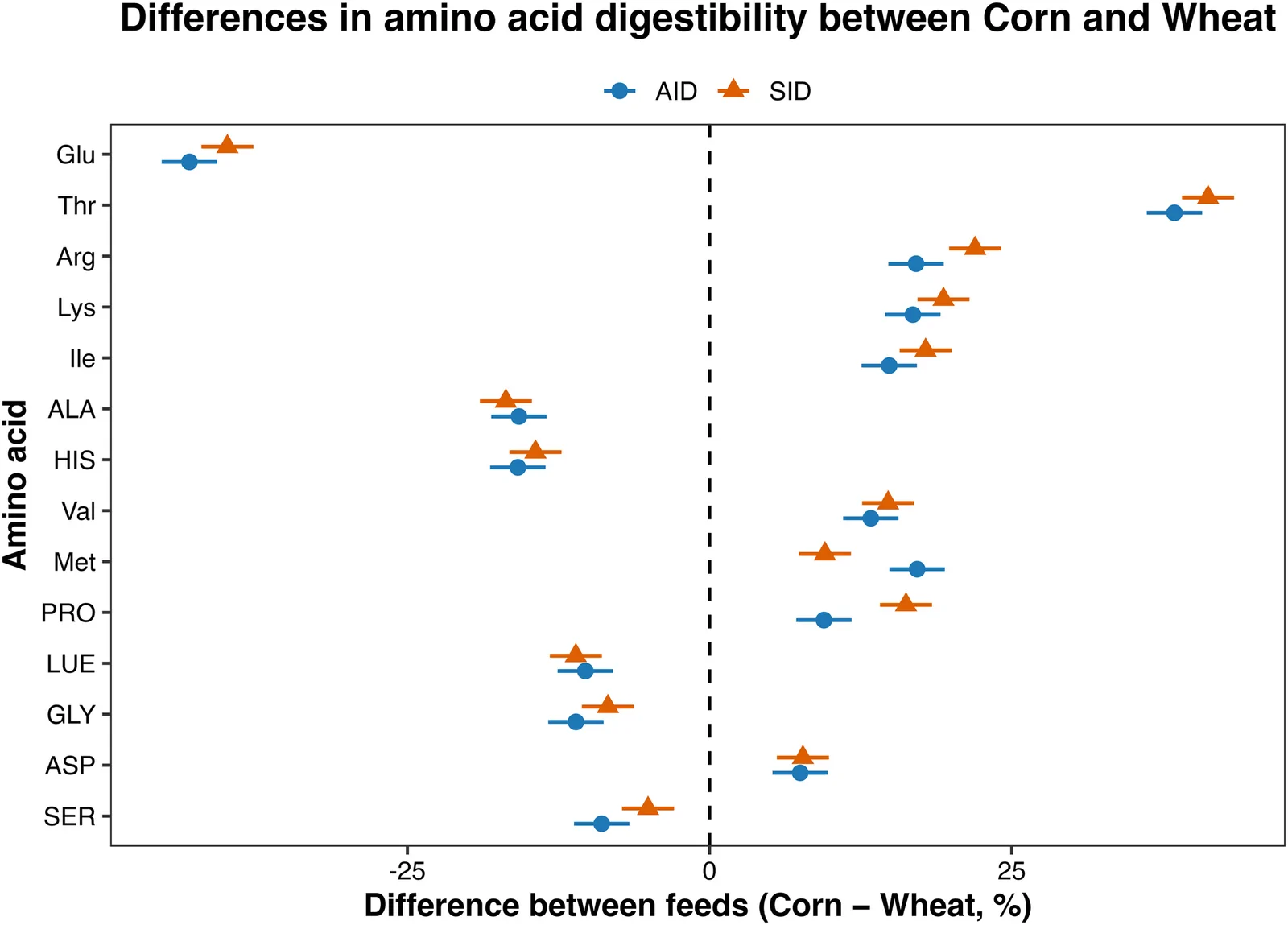
Differences in apparent ileal digestibility (AID; ●) and standardized ileal digestibility (SID; ▲) of amino acids between corn- and wheat-based diets. Values are presented as mean differences (Corn − Wheat, %) with 95% confidence intervals. Positive values indicate higher digestibility in corn, and negative values indicate higher digestibility in wheat. The dashed vertical line represents no difference between ingredients.

A limitation concern is the relatively low crude protein concentration of the corn-based test diet (6.34%) compared with the wheat-based diet (12.32%). Because the test ingredient was the sole source of dietary protein and amino acids, the lower protein concentration of corn resulted in lower concentrations of several amino acids in the corn-based diet. Low dietary amino acid concentrations do not necessarily introduce a systematic bias into the digestibility calculation when the dietary and ileal concentrations are accurately measured; however, they can reduce analytical precision because relatively small analytical or sampling errors represent a larger proportion of the measured amino acid concentration. Moreover, because standardized ileal digestibility is calculated by correcting AID using amino acid-specific basal endogenous losses, low amino acid concentrations can make the SID estimate more sensitive to uncertainty in the corresponding endogenous loss estimate. In addition, the substantially different crude protein concentrations of the two test diets should also be considered when interpreting between-ingredient differences. Because the present experiment was designed to estimate ingredient-specific digestibility rather than to compare isonitrogenous diets, differences in dietary amino acid concentration and the resulting sensitivity of SID calculations to basal endogenous loss estimates represent an additional source of uncertainty (Fig. 1).

In conclusion, the present study demonstrates that apparent and standardized ileal amino acid digestibility differs between the tested corn and wheat samples and among individual amino acids in 10–14-day-old Japanese quail. The principal contribution of this study is the provision of age-specific digestibility coefficients for the early starter period, extending previous measurements obtained in older quail. The results indicate that digestibility values should be interpreted in relation to both ingredient and bird age. The coefficients generated in the present study may provide useful inputs for formulation of starter diets for young Japanese quail, although their effects on growth performance and other production responses require confirmation in feeding studies.

## Author contributions

**Mehran Mehri:** Contributed substantially to the conception and design of the work, as well as the acquisition, analysis, and interpretation of data. He drafted the original manuscript and provided critical revisions for important intellectual content. Mehran reviewed and approved the final version of the manuscript and agrees to be accountable for all aspects of the work, ensuring that questions related to the accuracy or integrity of any part of the work are appropriately investigated and resolved.

**Mahmoud Ghazaghi:** Contributed substantially to the acquisition and analysis of data, including conducting laboratory analyses. He critically reviewed the manuscript for important intellectual content. Mahmoud reviewed and approved the final version of the manuscript and agrees to be accountable for all aspects of the work, ensuring that questions related to the accuracy or integrity of any part of the work are appropriately investigated and resolved.

**Mohammad Rookouei:** Contributed substantially to the conception and design of the work, as well as the interpretation of data. He critically reviewed the manuscript for important intellectual content and provided project administration. Mohammad reviewed and approved the final version of the manuscript and agrees to be accountable for all aspects of the work, ensuring that questions related to the accuracy or integrity of any part of the work are appropriately investigated and resolved.

## Declaration of competing interest

The authors declare that they have no known competing financial interests or personal relationships that could have appeared to influence the work reported in this paper.
